# Adjusted green HPLC determination of nirmatrelvir and ritonavir in the new FDA approved co-packaged pharmaceutical dosage using supported computational calculations

**DOI:** 10.1038/s41598-022-26944-y

**Published:** 2023-01-04

**Authors:** Mohamed S. Imam, Afnan S. Batubara, Mohammed Gamal, Ahmed H. Abdelazim, Ahmed A. Almrasy, Sherif Ramzy

**Affiliations:** 1 Pharmacy Practice Department, College of Pharmacy, Shaqra University, Shaqra, 11961 Saudi Arabia; 2 Clinical Pharmacy Department, National Cancer Institute, Cairo University, Cairo, Egypt; 3 Department of Pharmaceutical Chemistry, College of Pharmacy, Umm Al-Qura University, Mecca, 21955 Saudi Arabia; 4 Pharmaceutical Analytical Chemistry Department, Faculty of Pharmacy, Beni-Suef University, Beni-Suef, 62514 Egypt; 5 Pharmaceutical Analytical Chemistry Department, Faculty of Pharmacy, Al-Azhar University, Nasr City, Cairo, 11751 Egypt

**Keywords:** Drug discovery, Chemistry

## Abstract

The greening of analytical methods has gained interest in the quantitative analysis field to reduce environmental impact and improve safety health conditions for analysts. Nirmatrelvir plus ritonavir is a new FDA approved co-packaged medication developed for the treatment of COVID-19. The aim of this research was to develop green fitted HPLC method using pre experimental computational testing of different stationary phases as well as selecting mobile phase regarding to green analytical chemistry principles**.** Computational study was designed to test the physical interaction between nirmatrelvir and ritonavir and different columns (C8, C18, Cyano column). The study showed that the C18 column was better for simultaneous HPLC analysis of the cited drugs. Regarding to green point of view, mobile phase consisted of ethanol: water (80:20, v/v) provided an efficient chromatographic separation of nirmatrelvir and ritonavir within a short analytical run time, reasonable resolution and excellent sensitivity. Isocratic elution was performed on a selected C18 column and a green adjusted mobile phase at flow rate of 1 mL/min and UV detection at 215 nm. The chromatographic system allowed complete baseline separation with retention times of 4.9 min for nirmatrelvir and 6.8 min for ritonavir. The method succeeded to determine nirmatrelvir and ritonavir over the concentration range of 1.0–20.0 μg/mL in the pure form and in pharmaceutical dosage form. Greenness profiles of the applied HPLC method was assessed using analytical eco-scale, the green analytical procedure index and the AGREE evaluation method. The results revealed adherence of the described method to the green analytical chemistry principles. The authors hope to provide a promising challenge for achieving green goals through integrating computational tools and applying them with green assessment metrics.

## Introduction

Development of environmental friendly analytical methods is gaining researcher interest to reduce the environmental impact and improve safety health conditions for analysts. HPLC is a widely used method for analysis of drugs in various stages either manufacturing or confirming quality of bulk drugs and pharmaceutical formulations. HPLC methods usually recommend the presence of a hydrophobic stationary phase and a polar mobile phase to achieve an efficient separation process^1,2^.

Stationary phase representing different columns, C8, C18, Cyano type, is the main unit of HPLC recommended for separating different components existed in the same sample^3^. The ability to speed up HPLC runs enables fast analysis process and lower cost. Selecting an efficient column offers the chance for a good separation process and an acceptable chromatographic peaks.

Computational tools can help to decrease the number of possible experimental trials for different columns required for efficient chromatographic separation process. Integration of computational chemistry with pharmaceutical analysis provides assistive tool to predict physical and chemical properties of compounds. The computational chemistry generates data about structural properties complementary to experimental data and supports researchers with predictive results before running the actual experiments. The calculations are based mainly on quantum mechanics, molecular dynamics and semi empirical structure-properties relationships^4,5^. The density functional theory is commonly used approach to recognize the electronic structure of compounds. It can predict many molecular properties, molecular structures, vibrational frequencies, electrostatic interaction energies and binding energies between compounds^6,7^. Although many efforts have been made to optimize chromatographic compounds, little attention has been paid for computational studies use for testing the strength of physical interactions between various compounds and available columns^8,9^.

Mobile phase is usually a mixture of water (containing additives or buffer solutions to adjust pH) and organic solvent, as acetonitrile and methanol^10^. Acetonitrile and methanol are the common organic solvents used in HPLC regarding to complete miscibility with water, low viscosity and low chemical reactivity with compounds, the instrument components and the column surfaces^10,11^. Unfortunately, acetonitrile and methanol had undesirable impact on the environmental and health safety. As large amount of organic solvents can generate disposal waste, developing environmental friendly HPLC methods has received researcher interest in the analytical laboratories to find environmental friendly mobile phases and replace polluting analytical methods with cleaner ones^12,13^. The environmental friendliness of various organic solvents is evaluated based on environmental, health and safety criteria and life cycle analysis^13^. Ethanol is one of the most environmentally friendly organic solvents, which makes it highly desirable for environmentally friendly liquid chromatography^14^. Compared to acetonitrile and methanol, ethanol is less toxic and has a lower vapor pressure, which results in less evaporation and thus less inhaled amount. In addition, compared to acetonitrile and methanol, ethanol has lower disposal costs in terms of environmental impact^15,16^.

**Figure 1:**
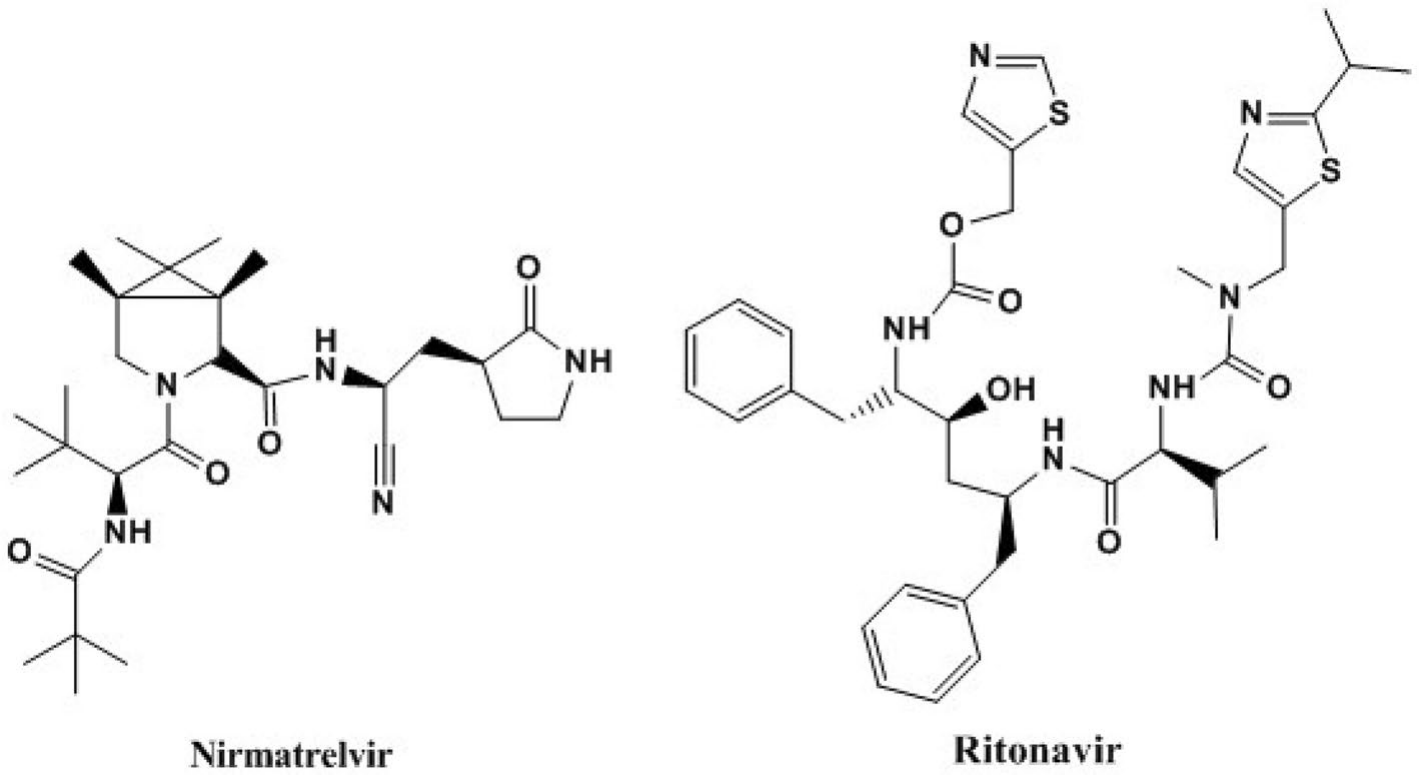
Structural formula of nirmatrelvir and ritonavir.

Nirmatrelvir plus ritonavir, Fig. 1, is a new combination medication approved in US, United Kingdom and Europe and for the treatment of COVID-19 in adults who do not require supplemental oxygen and are at high risk to severe COVID-19^17–19^. Nirmatrelvir is (1R,2S,5S)-N-((1S)-1-Cyano-2-((3S)-2-oxopyrrolidin-3-yl) ethyl)-3-((2S)-3,3-dimethyl-2-(2,2,2-trifluoroacetamido) butanoyl)-6,6-dimethyl-3- azabicyclo (3.1.0) hexane-2carnnboxamide. Its molecular weight is 499.54 and its molecular formula is C_23_H_32_F_3_N_5_O_4._ Nirmatrelvir is white to pale colored powder with practical solubility in methyl isobutyl ketone, 1-butanol and isopropyl acetate, sparingly soluble in anisole, n-propyl acetate, n-butyl acetate and insoluble in heptane^20,21^. Nirmatrelvir binds SARS-CoV-2 Mpro, also referred to as 3C-like protease or nsp5 protease, active site causes selectively and reversibly inhibiting of SARS-CoV-2 Mpro activity, with potent antiviral effect against several human coronaviruses, including SARS-CoV-2, SARS-CoV, and MERS. It has the ability to render the protein incapable of processing polyprotein precursors which leads to the prevention of viral replication^22^.

Ritonavir is 5-Thiazolylmethyl [(αS)-α-[(1S,3S)-1-hydroxy-3-[(2S)-2-[3-[(2-isopropyl-4-thiazolyl) methyl]-3-methylureido]-3-methylbutyramido]-4-phenylbutyl] phenethyl] carbamate. Its molecular weight is 720.94 and its molecular formula is C_37_H_48_N_6_O_5_S_2_. Ritonavir is white or almost-white powder with practical solubility in methanol, methylene chloride and sparingly soluble in acetonitrile^20,21^. Ritonavir is a protease inhibitor and CYP3A inhibitor of human immunodeficiency virus type 1. It is not active against SARS-CoV-2 Mpro. It inhibits the CYP3A-mediated metabolism of nirmatrelvir providing increased plasma concentrations of nirmatrelvir^23,24^.

Reviewing literature, LC–MS/MS was the only reported method for analyzing nirmatrelvir and ritonavir in human plasma^25^. To date, no analytical methods enabled determination of nirmatrelvir and ritonavir in the new FDA approved co-packaged dosage form. The purpose of this research was to develop an environmentally friendly HPLC method by preliminary testing different stationary phases, C8, C18 and Cyano columns, in advance and selecting the mobile phase considering the principles of green analytical chemistry. Furthermore, the green fitness of the applied HPLC method was assessed using the analytical eco-scale, the green analytical procedure index and the AGREE evaluation method. The proposed method showed superiority and agreement with the greenness characteristics in terms of the common green metric values. The authors hope to provide a promising challenge for achieving green goals by integrating computational tools and applying them with green assessment metrics.

## Experimental

### Materials and chemicals

Pure reference standard of nirmatrelvir (99.36%) and ritonavir (99.62%) were kindly supplied by Pfizer, Inc, Egypt.

Paxlovid tablets, pink oval nirmatrelvir film-coated tablet (150 mg) co-packaged with white ritonavir film-coated tablet (100 mg), (B. NO: A1324, manufactured by Pfizer Company), were kindly supplied by Pfizer, Inc., Egypt. The recommended dose is two tablets of nirmatrelvir plus one tablet of ritonavir.

Ethanol, HPLC grade was supplied by Sigma Aldrich, Germany. Water used throughout the procedure was freshly distilled.

### Apparatus

HPLC, LDC Analytical, Milton Roy, USA, equipped with diode array UV–visible detector and autosampler injector. Chromatographic analysis was performed using the data analysis program (Thermo ChromQuest 4.2.34, version 3.1.6).

### Standard solutions

Standard stock solutions of nirmatrelvir and ritonavir (100 μg/mL) were prepared by dissolving 10 mg of the drug powder in 50 mL of ethanol using a 100 mL volumetric flask and completing to volume with ethanol. Working concentrations were prepared by dilutions with the mobile phase (ethanol: water (80:20, v/v)).

### Procedures

#### Computational calculations to test the strength of physical interactions between nirmatrelvir and ritonavir with different columns

Nirmatrelvir, ritonavir, the major unit of C18, C8, and Cyano columns, as well as the corresponding complex products were created and optimized using Gauss-view software. The optimized products energy values were assessed. Density functional theory method at the B3LYP/6-31G (d) basis set level was used for the described calculations. The binding energy of nirmatrelvir, ritonavir with different columns was assessed using the following equation^4^:

$$\Delta {\text{E}} = {\text{E}}_{{{\text{A}} - {\text{B}}}} - {\text{E}}_{{\text{A}}} - {\text{E}}_{{\text{B}}}$$

Where A is the energy of the molecular structure of the nirmatrelvir or ritonavir, B is the energy of the molecular structure of column units and ∆E is the binding energy.

### Chromatographic conditions

An isocratic chromatographic procedure was lunched using BDS Hypersil C18 column (250 × 4.6 mm, 5 μm particle size) and ethanol: water (80:20, v/v) as a mobile phase. The mobile phase was degassed and pumped at a flow rate of 1 mL/min. 20 µL of the standard solution was injected and the detection was done at 215 nm.

### Construction of calibration graphs

Accurately measured volumes of nirmatrelvir and ritonavir working standard solutions were added to a series of 10 mL volumetric flasks to produce different solutions with concentrations ranging from 1.0–20.0 ug/mL of nirmatrelvir and ritonavir, then the volume was made up with ethanol. Isocratic elution of 20 µL aliquots with the mobile phase was done. Peak area values of nirmatrelvir and ritonavir were plotted against drug concentrations (ug/mL) and the calibration plots were obtained. In general, all methods were carried out in accordance with relevant guidelines and regulations.

### Procedures for tablets

Two unit of nirmatrelvir pharmaceutical tablets and one unit of ritonavir pharmaceutical tablet were scratched, weighed, and powdered. The prepared powder was added to 100-mL volumetric flask. The volume was made up to 50 mL with ethanol. The solution was shaken vigorously for 20 min, then sonicated and filtered. The volume was made up to 100 mL with ethanol to prepare a stock solution containing 3 mg/mL nirmatrelvir and 1 mg/mL ritonavir. Different working solutions were prepared with the mobile phase to obtain different concentrations of nirmatrelvir and ritonavir. The working standard solution was assessed by applying the procedure as previously described and the corresponding concentration was calculated from the regression equation.

## Results and discussion

Development of environmental friendly analytical methods is becoming highly desirable and attractive approaches for the researchers. Lunching green analytical HPLC method focuses mainly on using an efficient column, decreasing mobile phase consumption and replacing hazardous solvents, acetonitrile or methanol, by an environmentally friendly alternative.

Nirmatrelvir plus ritonavir is a new FDA approved co-packaged medication developed for the treatment of COVID-19. Regarding to recent lunch, there is a need to develop analytical method to provide feasible determination of nirmatrelvir and ritonavir in the bulk and pharmaceutical dosage form.

In the recent scientific work, the strategy was to develop an environmentally friendly HPLC tool and reduce the undesirable effects of hazardous solvents by using environmentally friendly alternatives. This strategy was implemented through a preliminary study of various available columns using computational calculations. In addition, ethanol was used as the main component of the mobile phase instead of methanol or acetonitrile. The HPLC method described allowed rapid simultaneous determination of nirmatrelvir and ritonavir in bulk and pharmaceutical form.

### Method development and optimization

To develop an efficient chromatographic procedure, several factors were carefully verified to assign the appropriate parameters enabled simultaneous determination of nirmatrelvir and ritonavir.

### Elution mode choice

Isocratic elution mode was chosen regarding to simplicity, cost effective and no need for re-equilibration of the column through repeating injections^26^.

### Pre experimental testing of different columns using computational calculations

The ability to speed up HPLC runs enables fast analysis process and lower cost. An efficient column helps to obtain good chromatographic process and acceptable separation peak. Hydrophobicity is the main mechanism for interaction between compounds and the various stationary phases. Nirmatrelvir and ritonavir are two drugs that varies in the hydrophobic and hydrophilic nature. Ritonavir is a very hydrophobic structure which can retain in the stationary phase longer than nirmatrelvir. On the other hand, nirmatrelvir is relatively hydrophilic which can retain shorter in the stationary phase. For fast analysis process, column choice was done using computational calculations. Density functional theory presents the continent tool to recognize the electronic nature of compounds. It helps to assign an actual characters of the compound based on electron density assessment^6,7^. The calculated binding energy for the complexes formed between nirmatrelvir and different columns were − 11.3, − 134.5, and − 32.4 kJ/mol for the cyano, C8, and C18 moieties, respectively. Also, the calculated binding energy for complexes formed between ritonavir and different columns was − 10.6, − 153.7, and − 50.4 kJ/mol for the mentioned columns, respectively. This result indicates that nirmatrelvir and ritonavir might have a strong affinity for the C8 column, which produces long analysis time. In addition, the calculated binding energy between nirmatrelvir and ritonavir was relatively close with the Cyano column, which indicated that the chromatographic peaks could not be well resolved and negatively impacted the resolution and symmetrical pattern of the obtained peaks. In addition, the calculated binding energy of nirmatrelvir and ritonavir with the C18 column showed relative variations in the obtained energy of the above drugs with the C18 column, indicated that the chromatographic peaks could be symmetrical and well separated. The high degree of hydrophobic properties of C18 column makes the retention time longer for the non-polar compounds, Ritonavir, provides an efficient process and reduces the tailing of the obtained peaks.

### Mobile phase composition

The nature of the mobile phase, especially the polarity, plays essential role in the selectivity of the chromatographic method. Recently, the choice of mobile phase is strongly recommended considering the health and environmental aspects of organic solvents^27,28^. The selection of mobile phase was based on the ranking of solvents in terms of green analytical chemistry metrics^29^. Ethanol was considered one of the environmentally friendly organic solvents, which made it a particularly desirable for green liquid chromatography^14–16^. In isocratic elution, different ratios of ethanol and water were checked to assign the main composition of the mobile phase. The experimental trials revealed that use of water in higher ratios than ethanol resulted in nirmatrelvir and ritonavir peaks appeared after a longer period and longer analytical run time. It could be explained by insufficient elution efficiency regarding to the low ethanol content. Therefore, increasing the ethanol content was recommended to provide an optimum chromatographic conditions. After extensive experimentation, a mixture of ethanol and water (80:20, v/v) proved to be the best mobile phase, providing efficient chromatographic separation of nirmatrelvir and ritonavir with short analysis run time, adequate resolution, and excellent sensitivity.

### Choice of appropriate wavelength

**Figure 2:**
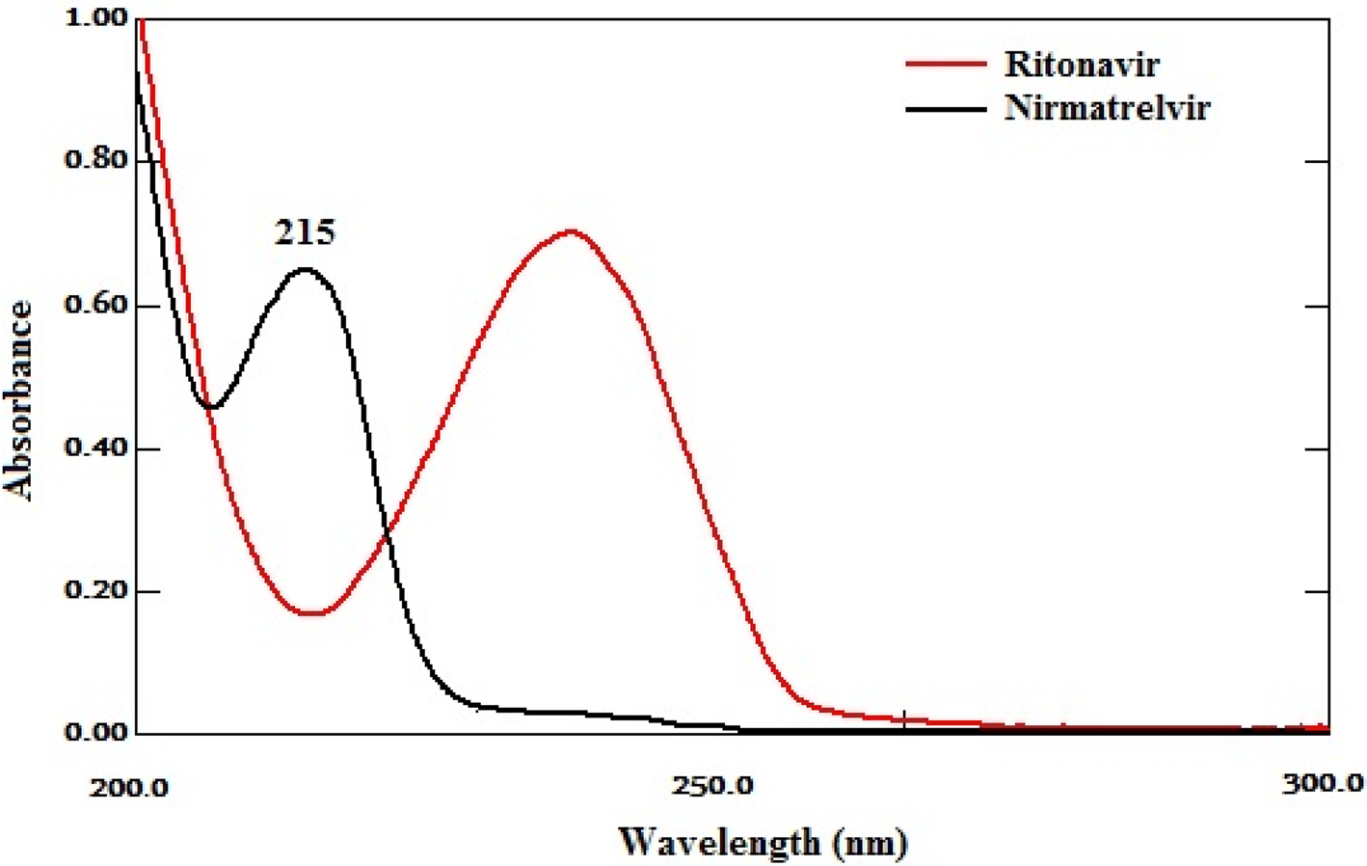
UV spectra of nirmatrelvir (15 μg/mL) and ritonavir (15 μg/mL).

After checking the UV spectra of nirmatrelvir and ritonavir, Fig. 2, together with testing different wavelengths, 215 nm was selected as it provided good sensitivity for nirmatrelvir and ritonavir.

### Flow rate of the mobile phase

The effect of the mobile phase flow rate on the chromatographic process of the drugs studied was verified. A flow rate of 1 mL/min represented an efficient chromatographic process within a reasonable time. It was found that a flow rate of 1.5 mL/min resulted in faster elution of nirmatrelvir and ritonavir, with narrow peak shapes developing. In addition, the peak of the first compound eluted, nirmatrelvir, was relatively close to the peak not obtained, resulting in an unacceptable value of the capacity factor and the lowest efficiency of the separation process. A flow rate of 0.5 mL/min provided acceptable chromatographic parameters but a longer analytical run time.

### System suitability testing parameters

**Figure 3:**
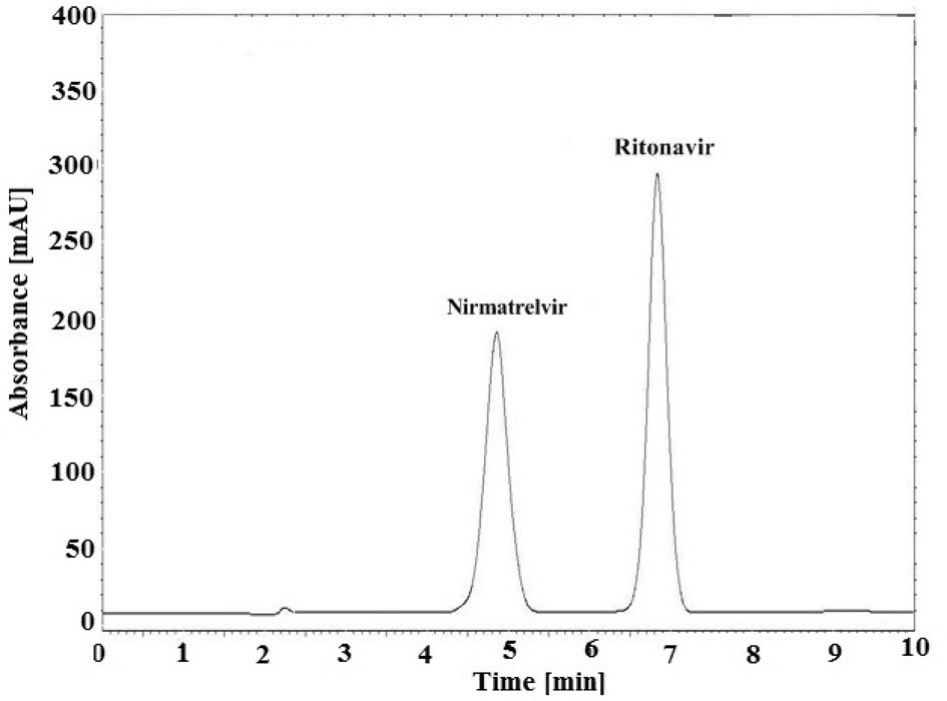
HPLC chromatogram of nirmatrelvir (15 μg/mL) and ritonavir (15 μg/mL).

In accordance with USP, the number of theoretical plates (N), resolution factor (Rs), retention factor (k), and tailing factor (T) were verified. The results, Table 1, revealed that the chromatographic system functioned properly during the analytical procedures. The resolution value (Rs) between nirmatrelvir and ritonavir peaks was calculated and found to be > 2. Moreover, the obtained values of retention factor, tailing factor and theoretical plates showed the acceptability of the proposed chromatographic procedure. The chromatographic peaks were better defined and resolved without tailing. The chromatographic system allowed complete baseline separation with retention times of 4.9 min for nirmatrelvir and 6.8 min for ritonavir, Fig. 3.

**Table 1:** Performance and characters of the described HPLC method for determination of nirmatrelvir and ritonavir.

Parameter	Nirmatrelvir	Ritonavir
Wavelength (nm)	215	215
Linearity range (μg/mL)	1.0–20.0	1.0–20.0
Coefficient of determination (r^2^)	0.9999	0.9998
Slope	165,873	318,082
Intercept	10,143	1929
LOD (μg/mL)	0.20	0.32
LOQ (μg/mL)	0.60	0.96
**System suitability parameters**		
Retention time (tR)	4.9	6.8
Retention factor (K´)	1.2	2.0
Theoretical Plates (N)	2533	2009
Tailing factor (T)	0.98	1.00
Retention time (tR)	4.9	6.8
Resolution (Rs)	3.8

### Method validation

The method described was validated in accordance with ICH guidelines regarding linearity, accuracy, precision, limits of detection [LOD], limits of quantitation [LOQ], and robustness.

### Linearity

The calibration plots were conducted under the described chromatographic conditions. Good correlation coefficients were obtained over the concentration range of 1.0–20.0 μg/mL, for nirmatrelvir and ritonavir. Regression parameters were summarized in Table 1.

### LOD and LOQ

LOD and LOQ were calculated based on the residual standard deviation of the regression line (SD) and the obtained slope using the following equations:

$${\text{LOD}} = 3.3\,{\text{SD}}/{\text{slope}}\quad \quad \quad {\text{LOQ}} = 10\,{\text{SD}}/{\text{slope}}$$

The results obtained, Table 1, revealed the sensitivity of the described method.

### Accuracy

The accuracy of the developed method was monitored by calculating the mean percent recovery (%R), for triplicate determination of three concentration levels of each drug (4.0, 8.0 and 16.0 µg/mL). The results obtained, Table 2, demonstrated the high reliability of the developed method.

**Table 2:** Evaluation of accuracy for determination of nirmatrelvir and ritonavir using the described method.

Nirmatrelvir			Ritonavir		
Taken (μg/mL)	Found (μg/mL)^a^	%R	Taken (μg/mL)	Found (μg/mL)^a^	%R
4.0	4.01	100.25	4.0	3.98	99.50
8.0	8.08	101.00	8.0	8.04	100.50
16.0	16.1	100.63	16.0	15.99	99.94
Mean ± %RSD		100.63 ± 0.375	Mean ± %RSD		99.98 ± 0.501

^a^Average of three determinations.

### Precision

The precision of the developed method was monitored by calculating the percent relative standard deviation (%RSD), for triplicate determination of three concentration levels of each drug (4.0, 8.0 and 16.0 µg/mL) within one day for repeatability and on three successive days for Inter mediate precision. The small values of %RSD demonstrated high precision of the proposed method as listed in Table 3.

**Table 3:** Precision study for the described method.

Parameters	Nirmatrelvir concentrations			Ritonavir concentrations		
	4.0 μg/mL	8.0 μg/mL	16.0 μg/mL	4.0 μg/mL	8 .0 μg/mL	16 .0 μg/mL
Repeatability	100.33	100.08	99.57	100.24	99.41	100.40
	101.25	100.25	98.67	100.25	100.91	101.25
	99.88	98.27	99.54	98.77	98.37	100.97
Mean ± %RSD	100.49 ± 0.698	99.53 ± 1.098	99.26 ± 0.511	99.75 ± 0.851	99.56 ± 1.276	100.87 ± 0.433
Intermediate precision	99.62	98.08	99.55	99.25	100.24	99.13
	98.71	100.05	100.33	98.79	100.91	98.01
	100.87	99.48	98.18	98.77	99.66	99.06
Mean ± %RSD	99.73 ± 1.084	99.20 ± 1.013	99.35 ± 1.088	98.93 ± 0.271	100.27 ± 0.625	98.73 ± 0.627

### Specificity

**Figure 4:**
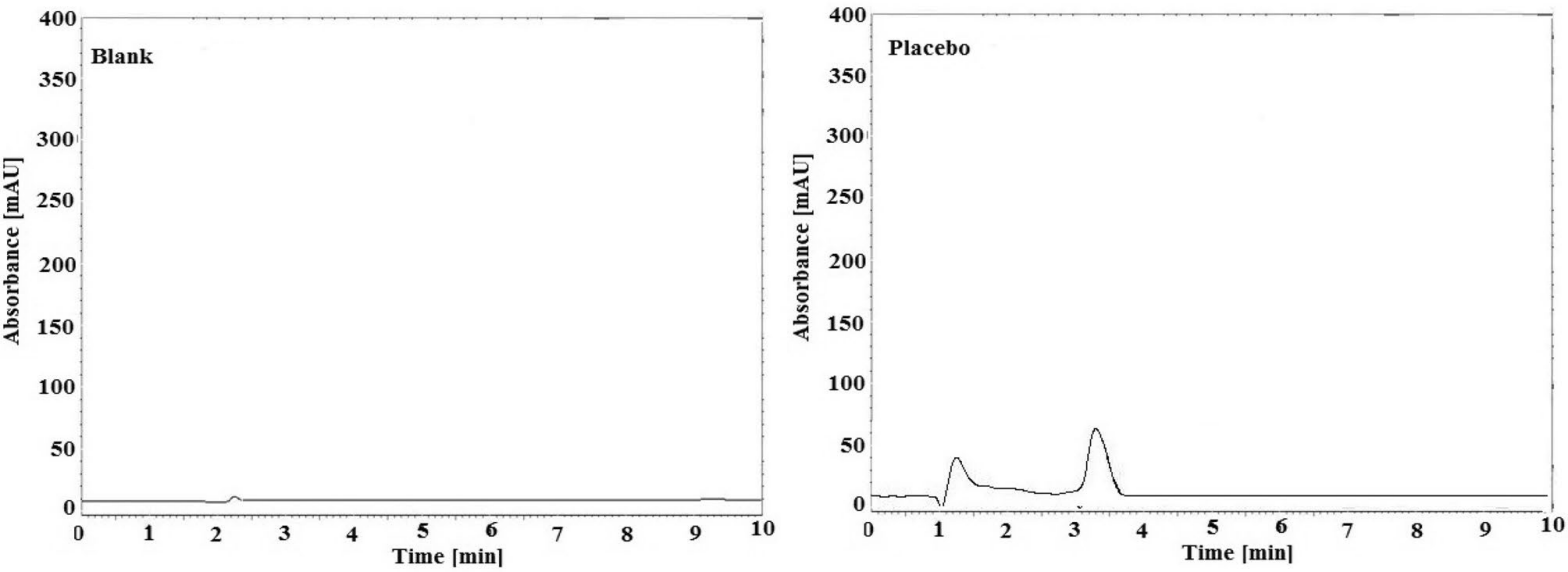
HPLC chromatograms of placebo and blank by the described method.

As the specificity of each method was confirmed by the selective ability to evaluate the compounds of interest in the presence of other components expected as matrix or blank compositions. Blank and placebo samples were prepared and tested to confirm that the retention times of the drugs under study did not interfere. The blank and matrix samples without active ingredients were assessed and compared with the chromatograms of nirmatrelvir and ritonavir, and no significant peaks were observed in the retention times of the investigated drugs. Only in the placebo chromatogram was a baseline ramp found at 3 min, but this did not affect the determination of the drugs studied. The chromatograms of blank and placebo using the described method are shown in Fig. 4. Moreover, method selectivity was achieved by estimation of nirmatrelvir and ritonavir in the presence of tablet matrix through standard addition technique. The results listed in Table 4 revealed that the method was sufficient selective for determination of the drugs under the study without interference from tablet excipients.

**Table 4:** Recovery study of nirmatrelvir and ritonavir by applying the standard addition technique.

Drug	Pharmaceutical taken, µg/mL	Pharmaceutical found, µg/mL	Pure added, μg/mL	Pure found, µg/mL	%R
Nirmatrelvir	5.0	4.94	3.0	3.01	100.33
			5.0	5.08	101.60
			7.0	7.11	101.57
Mean ± %RSD					101.16 ± 0.687
Ritonavir	10.0	9.94	4.0	4.03	100.75
			6.0	6.04	100.66
			8.0	7.95	99.38
Mean ± %RSD					100.26 ± 0.768

### Robustness

The robustness of the analytical method was tested by measuring its ability to remain unaffected by small but deliberate variations in the method parameters. In the described HPLC method, it was tested by minor changes in chromatographic conditions such as flow rate (± 0.1 mL/min) and mobile phase content ratio (± 2%). The described minor changes did not affect the resolution of nirmatrelvir and ritonavir, confirming the reliability of the described method as listed in Table 5.

**Table 5:** Robustness results for the determination of nirmatrelvir and ritonavir by the proposed HPLC method.

Parameters		Retention time (tR)		Tailing factor (T)		Resolution (Rs)
		Nirmatrelvir	Ritonavir	Nirmatrelvir	Ritonavir	
Flow rate (mL/min)	0.9	4.93	6.83	0.997	1.009	3.761
	1	4.90	6.80	0.984	1.003	3.755
	1.1	4.88	6.78	0.959	1.001	3.690
Mobile phase ratio (ethanol: water)	78:22	4.92	6.81	0.991	1.005	3.722
	80:20	4.90	6.80	0.984	1.003	3.755
	82:18	4.90	6.79	0.970	1.000	3.701

### Application of the described method for determination of the pharmaceutical preparation

The described HPLC method was applied to the determination of five prepared samples of nirmatrelvir, ritonavir, in paxlovid tablets. The obtained percentage recovery ± %RSD of the nirmatrelvir and ritonavir pharmaceutical tablets was 99.00 ± 0.83 and 99.40 ± 1.11 respectively. The results obtained revealed an agreement with the label claim and indicated feasibility of the described HPLC to be used in the quality control analysis of nirmatrelvir and ritonavir.

### Green evaluation of the proposed HPLC method

To assess the green adherence of the described analytical method, analytical eco-scale score^30^ was measured checking the amount of solvents consumed. Also, analytical eco-scale provides information reflecting the environmental impact of the analytical method. As the calculated score was 79, this revealed green excellence of the proposed method with prediction of minimal negative impact on the environment. Furthermore, green analytical methods index provides feasibility for check of different steps included in the described procedure. It considers sample preparation, sample handling as well as the chemicals consumed and the instrumentation. Every variable was colored from green to yellow to red indicating low, medium and high negative environmental impact respectively^31^. The applied HPLC method had five green zones and one red zone. Finally, AGREE tool^32^ was used, checking environmental friendliness profile of the analytical methods as a numerical value. The obtained value was 0.82 and confirmed the superior green characters of the developed HPLC method. The green assessment results were presented in Table 6.

**Table 6:** Green assessment results of the proposed HPLC method.

Tool applied	Data obtained
National environmental method index	
Green analytical procedure index	
The AGREE evaluation method	

## Conclusion

An adapted green HPLC method was developed for the determination of nirmatrelvir and ritonavir in pure form and in pharmaceutical dosage form. The method was developed using an assisted computational testing of the stationary phase as well as the green selection of the mobile phase was adopted. The evaluation of the environmental friendliness of the described method was performed using the analytical eco-scale, the green analytical process index and the AGREE evaluation method. The results showed that the HPLC method meets the environmental friendliness characteristics in terms of green metric principles.

## Data Availability

The datasets used during the current study are available from the corresponding author on reasonable request.

## References

[1] Koel M, Kaljurand M (2019). Green analytical chemistry.

[2] Ivanković A, Dronjić A, Bevanda AM, Talić S (2017). Review of 12 principles of green chemistry in practice. Int. J. Sustain. Green Energy.

[3] Kaczmarski K, Prus W, Kowalska T (2000). Adsorption/partition model of liquid chromatography for chemically bonded stationary phases of the aliphatic cyano, reversed-phase C8 and reversed-phase C18 types. J. Chromatogr. A.

[4] Cramer CJ (2013). Essentials of computational chemistry: Theories and models.

[5] Lewars, E. Computational chemistry. in *Introduction to the theory and applications of molecular and quantum mechanics* 318 (2011).

[6] Gross EK, Dreizler RM (2013). Density functional theory.

[7] Geerlings P, De Proft F, Langenaeker W (2003). Conceptual density functional theory. Chem. Rev..

[8] Attia KA, El-Abasawi NM, El-Olemy A, Abdelazim AH, El-Dosoky M (2018). Simultaneous determination of elbasvir and grazoprevir in their pharmaceutical preparation using high-performance liquid chromatographic method. J. Chromatogr. Sci..

[9] Attia KA, El-Abasawi NM, El-Olemy A, Abdelazim AH (2017). Application of an HPLC method for selective determination of phenazopyridine hydrochloride: Theoretical and practical investigations. J. AOAC Int..

[10] Snyder LR, Kirkland JJ, Dolan JW (2010). Introduction to modern liquid chromatography.

[11] Welch CJ (2010). Greening analytical chromatography. TrAC Trends Anal. Chem..

[12] Guideline IHT (2005). Impurities: Guideline for residual solvents Q3C (R5). Current Step.

[13] Yabré M, Ferey L, Somé IT, Gaudin K (2018). Greening reversed-phase liquid chromatography methods using alternative solvents for pharmaceutical analysis. Molecules.

[14] Płotka J (2013). Green chromatography. J. Chromatogr. A.

[15] Destandau E, Lesellier E (2008). Chromatographic properties of ethanol/water mobile phases on silica based monolithic C18. Chromatographia.

[16] Winterton N (2021). The green solvent: A critical perspective. Clean Technol. Environ. Policy.

[17] Lamb YN (2022). Nirmatrelvir plus ritonavir: First approval. Drugs.

[18] Hung Y-P (2022). Oral nirmatrelvir/ritonavir therapy for COVID-19: The dawn in the dark?. Antibiotics.

[19] Reina, J. & Iglesias, C. Nirmatrelvir plus ritonavir (Paxlovid) a potent SARS-CoV-2 3CLpro protease inhibitor combination. *Revista Espanola de Quimioterapia: Publicacion Oficial de la Sociedad Espanola de Quimioterapia* (2022). 10.37201/req/002.2022 PMC9134883 35183067

[20] Wanounou M, Caraco Y, Levy RH, Bialer M, Perucca E (2022). Clinically relevant interactions between ritonavir-boosted nirmatrelvir and concomitant antiseizure medications: Implications for the management of COVID-19 in patients with epilepsy. Clin. Pharmacokinet..

[21] Cokley JA (2022). Paxlovid^TM^ information from FDA and guidance for AES members. Epilepsy Currents.

[22] Yang KS, Leeuwon SZ, Xu S, Liu WR (2022). Evolutionary and structural insights about potential SARS-CoV-2 evasion of nirmatrelvir. J. Med. Chem..

[23] Lam C, Patel P (2022). StatPearls [Internet].

[24] Anastassopoulou C, Hatziantoniou S, Boufidou F, Patrinos GP, Tsakris A (2022). The Role of Oral Antivirals for COVID-19 Treatment in Shaping the Pandemic Landscape. J Pers Med..

[25] Martens-Lobenhoffer J, Böger CR, Kielstein J, Bode-Böger SM (2022). Simultaneous quantification of nirmatrelvir and ritonavir by LC-MS/MS in patients treated for COVID-19. J. Chromatogr. B.

[26] Schellinger AP, Carr PW (2006). Isocratic and gradient elution chromatography: A comparison in terms of speed, retention reproducibility and quantitation. J. Chromatogr. A.

[27] Rashed NS, Zayed S, Abdelazeem A, Fouad F (2020). Development and validation of a green HPLC method for the analysis of clorsulon, albendazole, triclabendazole and ivermectin using monolithic column: Assessment of the greenness of the proposed method. Microchem. J..

[28] Duan X (2020). A green HPLC method for determination of nine sulfonamides in milk and beef, and its greenness assessment with analytical eco-scale and greenness profile. J. AOAC Int..

[29] Prat D, Hayler J, Wells A (2014). A survey of solvent selection guides. Green Chem..

[30] Gałuszka A, Migaszewski ZM, Konieczka P, Namieśnik J (2012). Analytical eco-scale for assessing the greenness of analytical procedures. TrAC Trends Anal. Chem..

[31] Płotka-Wasylka J (2018). A new tool for the evaluation of the analytical procedure: Green analytical procedure index. Talanta.

[32] Pena-Pereira F, Wojnowski W, Tobiszewski M (2020). AGREE—Analytical GREEnness metric approach and software. Anal. Chem..

